# Pediatric Liquid Medications and Dental Caries: A Narrative Review

**DOI:** 10.1002/hsr2.71115

**Published:** 2025-07-23

**Authors:** Mohammed Taib Fatih, Mohammed Khalid Mahmood, Handren Ameer Kurda, Herve Tassery, Romain Lan, Delphine Tardivo, Mohammed Aso Abdulghafor

**Affiliations:** ^1^ Department of Dentistry Komar University of Science and Technology Sulaymaniyah Kurdistan Iraq; ^2^ Faculty of Medical and Paramedical Sciences Aix‐Marseille University, French National Center of Scientific Research (CNRS), French Blood Establishment (EFS), Bio‐Cultural Anthropology, Law, Ethics and Health Marseille France; ^3^ College of Dentistry Sulaymaniyah University Sulaymaniyah Kurdistan Iraq; ^4^ Dental School of Medicine, Conservative and Endodontic Department Aix‐Marseille University Marseille France; ^5^ Marseille Hospital APHM IHU‐MEPHI Institute Marseille France

**Keywords:** children, dental caries, dental erosion, oral health, pediatric liquid medications, sweetened medicines, syrups

## Abstract

**Background and Aims:**

Children with chronic conditions such as heart diseases, asthma, severe respiratory infections, epilepsy, organ failure, tumors, and recurring acute diseases such as sinusitis, otitis media, tonsillitis, or even iron and vitamin supplements are taking pediatric liquid medications (PLMs) for long durations. There is a growing concern and evidence in the literature about the harmful effects of PLMs on dentitions. This narrative review aims to provide a comprehensive update on PLM consumption as a risk factor for dental caries.

**Methods:**

An electronic search was conducted across various databases such as PubMed, Embase, Web of Science, Cochrane, Scopus and Google Scholar using the relevant MeSH terms and keywords “liquid medication,” “pediatric liquid medication,” “chronically ill children,” “children,” “dental caries,” “dental erosion,” “sugar content of medications,” and “endogenous pH of medications”.

**Results:**

A total of 33 studies were included in this narrative review. Some reports have linked the usage of PLMs in chronically ill children to dental erosion and caries. Physiochemical properties of PLM solutions, such as their sugar content and endogenous pH define their carcinogenicity and erosive potential. Indeed, several in vitro studies reported the erosive potential of PLMs on dental hard tissue structure when viewed under scanning electron microscopes. Some other studies have focused on the role of pediatricians, dentists, and families/parents.

**Conclusion:**

Studies support a positive association and suggest a higher caries risk experience among chronically ill children that consume PLMs. A multidisciplinary collaboration is needed between pediatricians, dentists, manufacturers and families to maximize the benefits of PLMs and minimizing its possible harms on oral health.

## 1 Introduction

Dental caries is a complex, multifactorial bacterial disease which results in the destruction of the dental hard tissue. The development of dental caries is primarily influenced by several factors, including the frequency of exposure to fermentable carbohydrates and dietary acids, the accumulation and retention of bacterial dental plaque, the availability of natural protective agents such as saliva, insufficient fluoride exposure, and most importantly, lack of proper dental hygiene measures [1]. Socioeconomic variables that have been inversely linked to early childhood caries (ECC) include the care provider's reported family income and educational level. Besides, nutritional status, malnutrition and serum vitamins have also been linked to dental caries [2, 3, 4, 5].

Pediatricians, physicians, and dentists prescribe medications for a variety of illnesses. There are various ways to provide medication, such as parenteral, cutaneous, sublingual, nasal, and oral. Oral administration is the most widely used and traditional method among these. Even when coated to hide the bitter tastes, oral drugs such as pills or capsules are unsuitable for use in children since they are too young to ingest them. Therefore, syrups—liquid medications—are the ones that are mostly recommended for use in youngsters. Syrups have been used in pediatric care for a very long time. These drugs have many benefits, such as high intestine absorption, easy availability and administration, flexible dosage, and widespread acceptance by children [6, 7, 8].

The usage of PLMs is usually for a short time; however long long‐duration treatment is needed in chronically ill children. For example, Children on PLMs for chronic conditions such as heart diseases, asthma, severe respiratory infections, epilepsy, organ failure, tumors, and recurring acute diseases such as sinusitis, otitis media, tonsillitis, or even iron and vitamin supplements are in need of PLMs for long durations [9].

Generally, short‐time consumption of PLMs is safe. However, there is a growing concern and evidence in the literature about the harmful effects of PLMs on dentitions. Some reports have linked the chronic usage of PLMs in chronically ill children to dental erosion and caries. If a disease lasts more than 3 months, the National Center for Health Statistics classifies it as chronic. Furthermore, taking medication on a daily or alternate day basis for longer than 3 months is considered long‐term [9, 10].

It is doubtless that chronically ill children need special care dentistry as they are more vulnerable to oral health problems. Defining the risk factors of this vulnerability has the utmost importance for its prevention and treatment. Hence, this narrative review aims to provide a comprehensive update on the relationship between PLMs and dental caries, with a particular emphasis on the physiochemical properties of PLMs—such as pH, sugar content, and acidity—and their potential role in promoting dental erosion and caries. Additionally, the review seeks to examine relevant in vitro, in vivo, and epidemiological studies, and to discuss the roles of pediatricians, dentists, and families in mitigating these oral health risks.

## 2 Methodology

**FIGURE 1:**
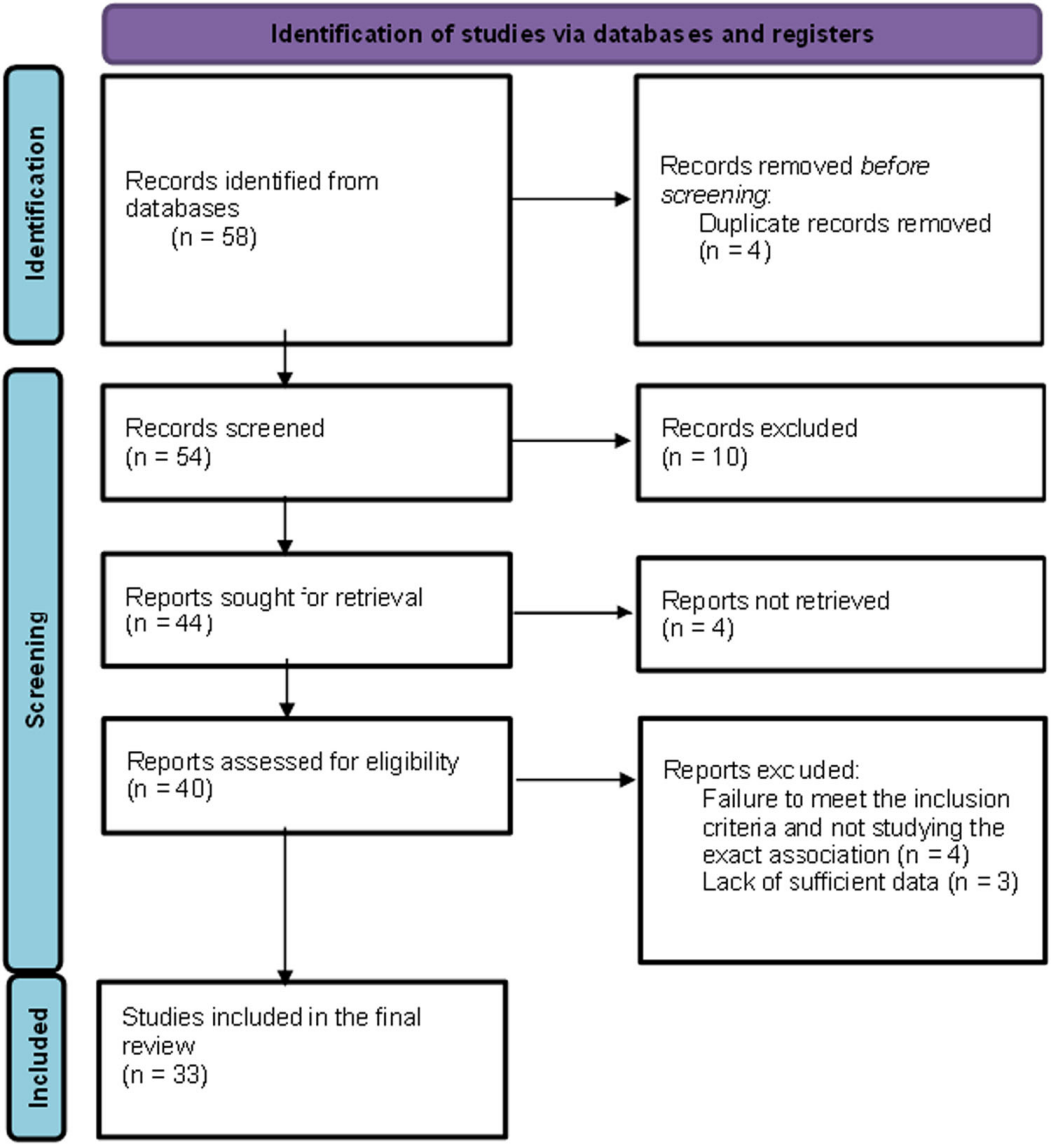
PRISMA flowchart of the records selection process.

In January 2023, an electronic search was conducted across various databases such as PubMed, Embase, Web of Science, Cochrane and Scopus using the relevant MeSH terms and keywords “liquid medication,” “pediatric liquid medication,” “chronically ill children,” “children,” “dental caries,” “dental erosion,” “sugar content of medications,” and “endogenous pH of medications.” The main search strategy applied was the following: (“pediatric liquid medication” OR “pediatric medication”) AND (“children” OR “chronically ill children”) AND (“dental caries” OR “tooth decay” OR “dental erosion”). All the preclinical/in vitro and clinical/epidemiological studies were included. Records that did not study the association or reported insufficient data on the association were excluded. To ensure maximum coverage, a further search was conducted on Google Scholar and the reference list of the previously identified records. The search had no date restriction; however, the language was restricted to publications only in English. The initial screening yielded 58 records. Only 40 reports were relevant to the topic of the review. Furthermore, seven records were excluded due to failure to meet the exact inclusion criteria. Finally, 33 papers were included in this narrative review. Figure 1 shows the flowchart of the study selection process. The included studies were thoroughly read, then classified into relevant sections. Included records were critically analyzed, and new synthesis was put forward to reach plausible conclusions.

## 3 Results

### 3.1 Physiochemical Properties of PLMs

Physiochemical properties of PLM solution define its carcinogenicity and erosive potential. In relation to dental caries, the most important physiochemical factors of PLMs are sugar content, endogenous pH, viscosity, titratable acidity, and soluble solid content. The following subsections will focus on these properties and their contribution to the initiation and progression of dental caries.

#### 3.1.1 Sugar Content

Sweeteners in pediatric medications usually affect compliance. Oral drugs with poor palatability may cause noncompliance in juvenile patients, affecting treatment success and efficacy. Drug producers add sweeteners and flavorings to hide their formulations' taste and smell to improve palatability [11].

Several studies have investigated the sugar content of PLMs around the world. For example, in Brazil, 59 PLMs were selected from the national health database, which belonged to 11 therapeutic classes. High sugar contents were identified in the antitussives (53.25%) and anticonvulsants (51.75%) [12]. Moreover, Al‐Batayneh et al. measured the sugar content of the 42 most commonly prescribed PLMs in Jordan. The sucrose mean was 8.1 for respiratory, 5.7 for CNS, 7.3 for antibiotics, 4.4 for gastrointestinal, and 35.3 for nutritional PLMs [13]. Valinoti et al. reported that out of 29 tested PLMs, 24 of them contained sucrose [14]. In an Indian research, out of 96, only four drugs displayed exactly the same sugar content as that on the content sticker. These four medications belonged to the nutritional supplement category [15]. While the consensus links high sugar content in PLMs to increased risk of dental caries, specific thresholds can aid clinical decision‐making. For instance, medications containing sucrose levels exceeding 10 g per dose are considered significantly cariogenic, especially with frequent administration. Studies have shown that many PLMs, particularly in certain therapeutic classes like antitussives and nutritional supplements, contain sucrose amounts well above this threshold [12, 13, 14, 15].

Recent developments have introduced sugar‐free formulations that replace sucrose with non‐cariogenic sweeteners such as sorbitol, xylitol, or artificial sweeteners. These alternatives typically contain less than 1 g of fermentable carbohydrate per dose, substantially reducing cariogenic potential. Multiple studies demonstrate that sugar‐free PLMs are equally effective in taste and compliance and pose a lower risk for dental caries [16].

Sugar and dental caries have a well‐established relationship. Oral bacteria, especially Streptococcus mutans, convert sugar into weak organic acids through metabolism. These acids lead to a pH drop below the crucial value of 5.5, which demineralizes enamel and advances the disease toward dental caries. Sugar content of PLMs may provide a substrate for the initiation of this process.

#### 3.1.2 Endogenous pH

Dental erosion is the irreversible damage produced by chemical processes like nonbacterial acids, and mechanical activity like abrasion and attrition. Intrinsic and extrinsic causes may cause dental erosion. Foods with high acidic content and beverages like sports drinks, wines, and medicines containing acid are linked to intrinsic factors, while extrinsic variables include acid reflux and vomiting owing to gastrointestinal diseases. Early erosion begins with enamel softening, which varies by acid type and contact time. Without appropriate intervention, erosion dissolves enamel crystals and damages the tooth structure permanently [17]. Dental erosion in preschoolers is studied internationally, and its prevalence ranges from 25% to 75% depending on the circumstances [18, 19].

PLMs are kept in an acidic state to expand their lifespan duration. Therefore, it is not rare for the PLM solutions to be acidic. Various researchers have measured the endogenous pH of PLMs. Many antipyretics and analgesic PLMs had features that can promote dental erosion [20]. For instance, Deshpande et al found that every drug class examined had an acidic pH, with anticonvulsants having the lowest mean (4.2) [15]. In a research investigating 42 PLM types, pH mean was lowest for nutritional class (3.8), followed by respiratory (4.1), gastrointestinal (4.7), CNS (5.1), and Antibiotics (6.4) [13]. In another research with 29 PLMs, 15 had a pH that was below the threshold for hydroxyapatite dissolution. Similar results were reported from Egypt as pH analysis revealed that all samples (15) were acidic, and 9 PLMs presented pH values below the critical value of 5.5 [21]. An alarming pH mean of 2.61 has been reported for antipsychotic PLMs [12]. Regarding the analgesics, almost all of the tested PLMs (93.8%) had pH values ≤ 5.5. Some studies have reported few PLMs having basic pH. Iron and calcium supplements were also basic in nature [15].

In conclusion, endogenous pH of PLMs contributes to dental caries in two ways: dental erosion of the enamel surface and reducing the dental plaque pH.

#### 3.1.3 Viscosity and Total Soluble Solid Content

Total soluble solid content measures the formulation's solid ingredient and directly affects viscosity. However, viscosity measures a fluid's internal friction and flow resistance. Generally, higher sugar content results in a higher viscosity and total soluble solid content. Viscous PLMs can reach fissures and toothbrush‐inaccessible regions. As they contain high levels of soluble solids and total sugars, anticonvulsants and antitussives were deemed risk factors for dental caries [12]. Viscosity values of all the tested PLMs were low and comparable except the antiepileptic Phenytoin [21]. The highest soluble solids values were found in bronchodilators [15].

**FIGURE 2:**
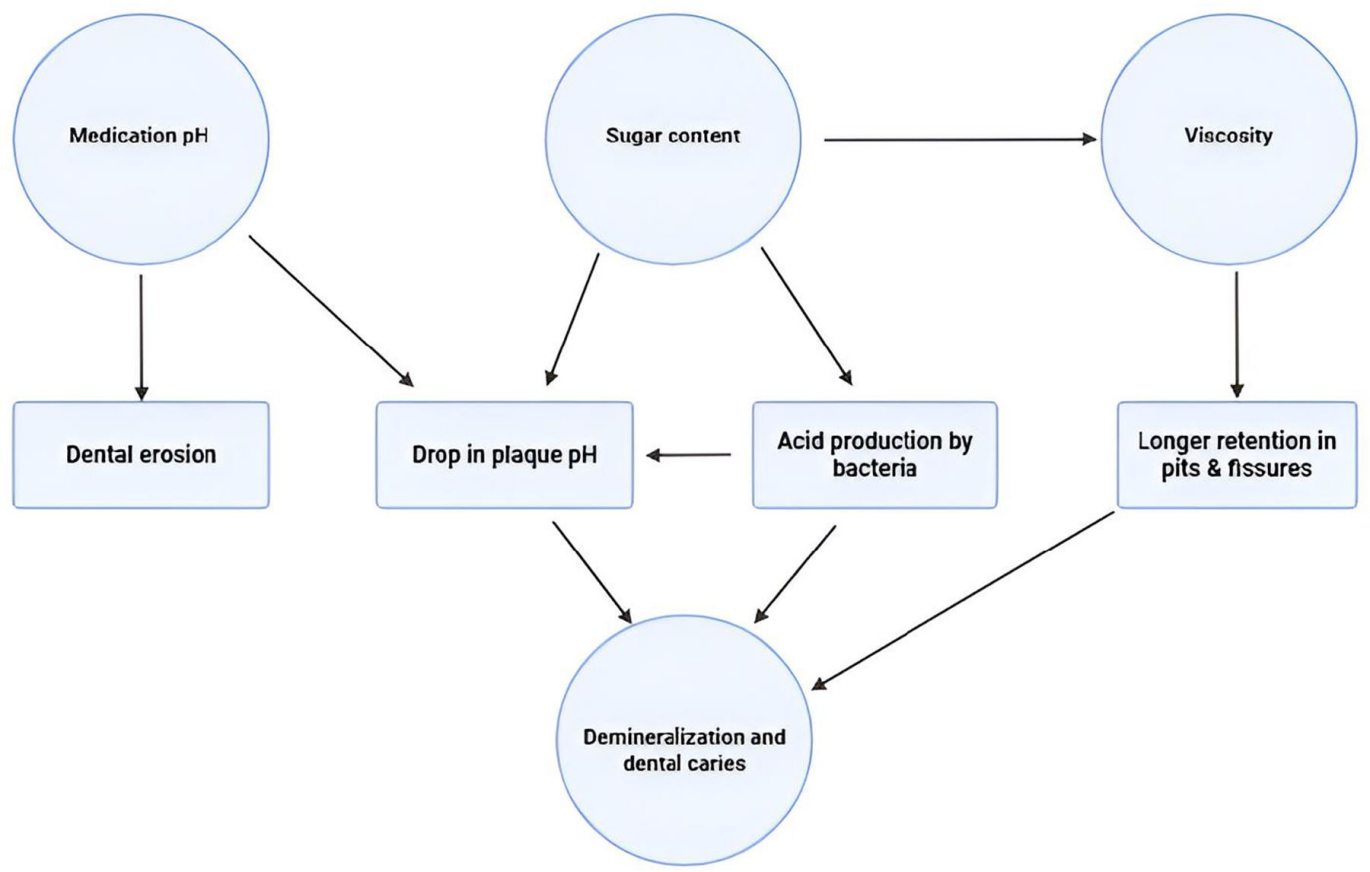
Contribution of physiochemical properties of pediatric liquid medications in dental caries.

Figure 2 illustrates the contribution of physiochemical properties of PLMs in dental caries.

### 3.2 In Vitro Studies

Normally, dental caries, especially once cavitated, is an irreversible disease. Therefore, true in vivo studies are unethical. Consequently, a relatively broad literature of in vitro effects of PLM solutions on tooth structure is present.

For example, all of the PLMs used during an investigation had an erosive effect on enamel. The majority of the drugs resulted in crater formation, irregular rod ends, and an etched prism pattern of erosion when examined under scanning electron microscopy (SEM) [22]. SEM images of respiratory, anti‐asthmatic, and iron‐containing PLMs showed structural degradation after 28 days, unlike those immersed in artificial saliva. Medicine type and exposure time affected deciduous enamel degradation [23]. Likewise, Time and immersion medium interaction showed that antitussives gradually decreased surface micro‐hardness on all days (7, 14, 21, and 28) [24]. Even iron and multivitamin drops have the potential to cause erosion and can affect the enamel micro‐hardness of primary teeth [25].

**FIGURE 3:**
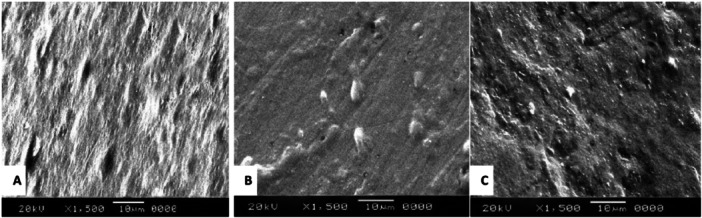
(A) Etched prism pattern after 10 min exposure to an antibiotic pediatric liquid medication. (B) Sporadic rod ends were seen under SEM after 1 min exposure to a multivitamin pediatric liquid medication. (C) Crater formation after 1 min exposure to antiepileptic pediatric liquid medications. Reproduced from [22].

**FIGURE 4:**
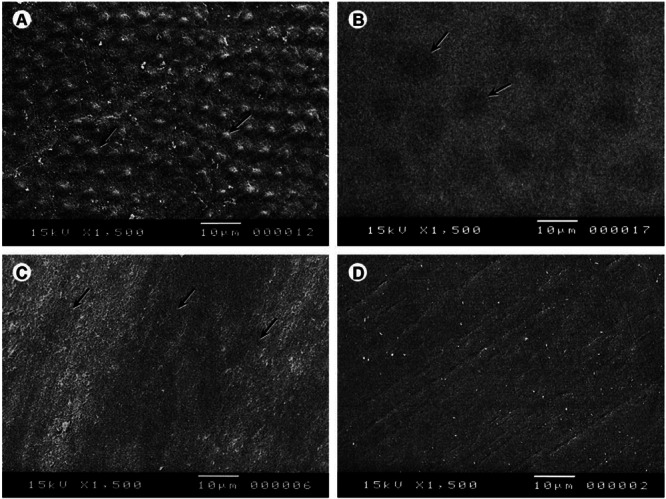
Scanning electron micrograph analysis of enamel structure after 28 days of immersion cycles in pediatric liquid medications. Arrows indicate the structural defects: (A) Respiratory expectorant; (B) ferrous sulfate; (C) salbutamol sulfate (anti‐asthmatic); D: artificial saliva. Reproduced from [23].

In a recent study conducted on over‐the‐counter PLMs, researchers concluded that even these kinds of medications that are generally regarded as safe could soften the enamel's surface and considerably reduce its micro‐hardness; this would result in damage to the enamel's surface as seen by SEM after multiple immersion cycles. It appears that oral liquids can erode deciduous tooth enamel, regardless of sugar or pH of PLMs [26]. In contrast, antibiotics killed *S. Mutans* without causing primary enamel mineral loss, regardless of sucrose content [27]. Figures 3 and 4 show the erosive potential of the PLM solution on the dental hard tissue structure.

In conclusion, findings from in vitro studies show that PLMs can affect the surface micro‐hardness and enamel roughness. In addition, PLM antibiotics reduce S. Mutans count in dental plaques.

### 3.3 In Vivo Studies

In vitro studies, no matter how perfectly organized, lack the intraoral environment, mainly the buffering and protective capacity of saliva and the presence of cariogenic bacteria. Various in vivo studies on the research question have been performed. For example, in an early research in 1981, Feigal and Jensen studied the carcinogenicity of seven commonly prescribed PLMs by detecting intraoral microbial plaque pH alterations after 30 min of rinsing the mouth with each medication. The intraoral pH response to various drugs was comparable or higher than sucrose rinses alone [28]. Furthermore, Mentes assessed the effects of sugared and sugar‐free PLMs on dental plaque pH after 1 h of ingestion. Minimum pH values (sugar‐free: 5.62 ± 0.36; sugared: 5.00 ± 0.33, *p* < 0.001) and maximum pH drops (sugar‐free: −0.57 ± 0.26; sugared: −1.16 ± 0.44, *p* < 0.001) were significantly different [29]. Sunitha et al. measured the drop in the plaque pH in six commonly used PLMs. They found that compared to the mean drop of plaque pH of sucrose solution (10%), half of the drugs had a significant drop between the baseline and 30 min after consumption [30]. In a relatively recent research, Naik et al. investigated the pH drop after rinse with 10 PLMs (analgesics, antibiotics, anti‐asthmatics, and multivitamins). Most of the individuals had a significant decrease in plaque pH, with the greatest pH drop occurring 5 min after PLM rinsing. Within 60 min, there was a steady return to the pH close to normal [31].

Findings from in vivo studies support the in vitro experiments, as they suggest that internal plaque pH drops after rinsing/drinking PLM solutions and the effect is directly proportional with the sugar content.

### 3.4 Epidemiological Studies

Epidemiological studies analyze disease rates and causes in diverse populations. Epidemiological data are used to plan and assess disease prevention and treatment programs. Epidemiology, like clinical observations and pathology, is essential to disease description.

In an epidemiological context, several studies have been performed among chronically ill children. In an early research in 1979, Roberts & Roberts compared 44 children under the age of six who took PLMs regularly for 6 months with similar‐aged youngsters. Mean decayed and filled score for the case group was 5.55, while the mean for the control group was 1.26. Children taking sucrose‐based medications had more carious teeth [32]. Moreover, intake of nocturnal medication in asthmatic patients (4–5 years of age) was associated with higher dental caries measured by dmft index compared to the controls (OR = 2.41) [33]. Furthermore, 455 children aged 2–12 with varied chronic conditions got PLMs for more than 6 months compared to 531 children of comparable age and diseases who received other medications. Children on PLMs had a higher risk of dental caries compared to those on other drugs (OR: 3.142, *p* < 0.001). Long‐term use of PLMs containing sucrose was associated with tooth decay in chronically ill children (77.8%) compared to other medications (52.7%) [34].

In a research concerning epileptic children, 84 epileptic children aged 2–12 using PLMs for more than 3 months were compared to 106 children of similar age and disease on other medications. Children taking PLM had a 2.55% higher incidence of dental cavities than those taking other drugs. Caries prevalence was higher in the study group (76.1%) than in the control group (55.6%). Children with epilepsy who used sucrose‐containing liquid medications for long periods were at higher risk for dental caries [35]. However, in 119 children with neuropsychomotor disorders, Brazilian researchers found no association between dmft/DMFT and medication consumption, duration, night medication, or sucrose‐containing medicine. There was no statistically significant link between sucrose‐containing continuous medication and dental caries, suggesting that other risk factors contribute to the condition [36].

In short, most of the epidemiological studies point out the fact that chronically ill children on continuous PLMs are at greater risk of dental caries compared to healthy children or sick children but on different types of medications.

### 3.5 Role of Pediatricians

The dental literature increasingly emphasizes that physicians should begin risk‐factor determination, preventative counseling, and prophylactic therapies in the first year of life to prevent dental caries. As they see patients for well‐baby visits and advise parents and caregivers, pediatricians can start this process. Pediatricians may help patients who can't afford dental care to improve their dental health. In the special context of chronically ill children on PLMs, knowledge, attitude, and practice certainly affect the oral health of these patients [37].

Various surveys have been implemented to measure the awareness and practice of physicians and pediatricians on the subject. For instance, only 50% of physicians knew that drugs without added sugar were available. The majority of pediatricians believed that prescriptions without added sugar are more costly and less sweet than those with added sugar [16, 38]. In fact, sugar‐free medications are sweet since they are made using non‐acidogenic and non‐cariogenic sweeteners like sorbitol, mannitol, and xylitol. Pediatricians were aware of the hidden sugars in PLMs, but very few of them informed parents about the risks of dental caries associated with PLMs and recommended oral hygiene practices [39].

In a survey from Turkey, after writing a prescription, 54.3% of the respondents educated their patients on dental health. Merely 27% of the pediatricians thought they were knowledgeable enough to advise parents about dental hygiene following drug use. The majority of participants (42.7%) and (50.7%) did not know that PLMs and dental erosion and caries formation are related, respectively [40]. Similar results were reported by other research internationally [41, 42, 43]. Finally, in a national survey conducted in the United States, 55% of participants said they had trouble in successfully referring their uninsured patients to dentists, while 38% said they had trouble referring their patients with insurance [37].

In conclusion, surveys suggest that pediatricians and physicians, who are in charge in prescription of PLMs for chronically ill children, are aware of the association between sugar and dental caries; however, this awareness is decreasing when it comes to the acidity of the PLMs. In addition, while the majority of pediatricians agree that physicians should teach families about oral hygiene and screen kids for dental caries, many also mention feeling untrained and lacking confidence.

### 3.6 Role of Parents and Families

A child's ability to maintain healthy teeth is greatly influenced by their family, specifically their parents' opinions toward the value of oral hygiene. The development of children's oral hygiene habits and the incidence of oral diseases can be influenced by parental skills and views on oral hygiene. Furthermore, studies have shown a clear correlation between children's dental health with family income and parental education [2].

Some surveys have been conducted on the knowledge, attitude, and practice of families and parents concerning the PLMs. For instance, in a particular survey, 63% of mothers gave children their medications twice a day, and 80% of them preferred the syrup form. None of the mothers cleaned the teeth of their children after taking medication. Approximately 79% of the mothers were not aware of the connection between caries and PLMs [44]. There is a lack of parental awareness of PLM use, and frequent PLM use negatively impacts children's dental health, particularly in high‐risk patients such as children with chronic diseases [45]. In another questionnaire assessment, of the mothers, 43.2% acknowledged a link between PLM usage and dental cavities or problems in tooth structure, and 40.7% of them said this was because the formulations contained sugar. After the child consumed medicines, just 29% of the participants gave them dental hygiene instructions, despite the fact that 81% had never been told how important this practice was [46]. Furthermore, although many parents are aware that sugar can lead to tooth decay, they typically do not connect this phenomenon to the additional sugar in PLMs [29, 46].

In short, surveys among parents on the possible harmful effects of PLMs on children's dentition reveal a necessity of raising their awareness on the subject by dentists and physicians, especially concerning the preventive measures after PLM consumption.

### 3.7 Role of Dentists

Dentists are the pioneers in treating and preventing dental cavities, and modern dentistry is focused more on preventative care than on surgical treatment. It is already well known that patients' dentists might have an impact on their oral hygiene‐related behaviors. For every patient, dentists need to do a dental caries risk assessment. Caries risk assessment is defined as “the determination of the likelihood of the incidence of caries during a certain time period or the likelihood that there will be a change in the size or activity of lesions already present” by the American Academy of Pediatric Dentistry [47].

Dentists should be aware of the potential oral consequences of PLMs, particularly in children with chronic illnesses, and take it into consideration as a potential risk factor for dental caries. Additionally, dentists must take an active role in the medical teams that treat children with persistent diseases. Despite these facts, authors of this review could find no articles in the literature on the knowledge, attitude, and practice of dentists and pediatric dentists on the research question.

### 3.8 Interparty Collaboration

**FIGURE 5:**
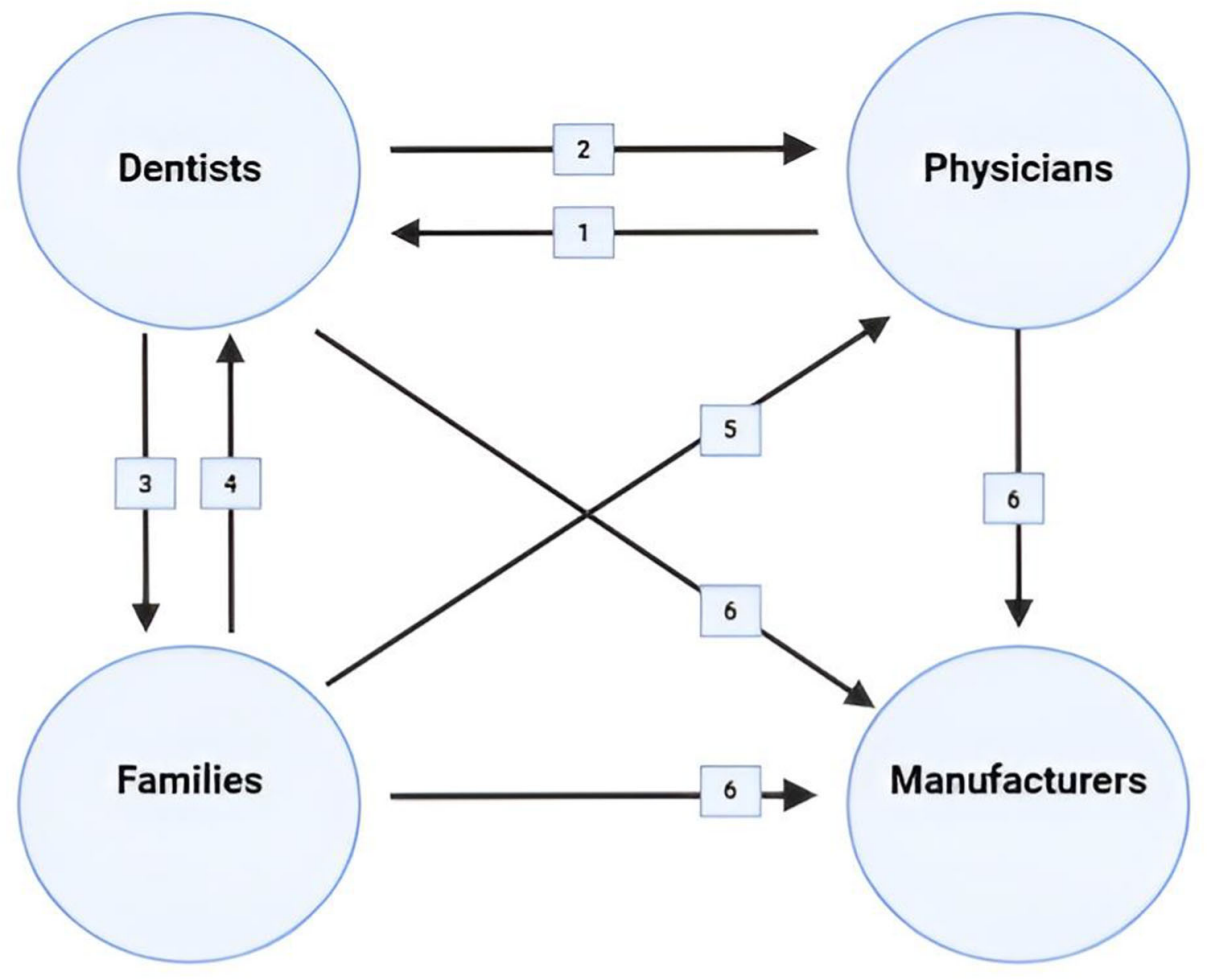
Proposed collaboration between parties to minimize the potential harms of PLMs on the oral health of children. 1. Dentists help physicians to choose the best‐tailored medication with the least harm to oral health. 2. Physicians and pediatricians refer children on chronic PLMs to be monitored regularly by dentists. 3. Dentists instruct families about the potential impact of PLMs on dentitions and raise their awareness toward preventive measures. 4. Families regularly consult dentists to minimize the risks. 5. Families demand for alternative dentition‐friendly medications. 6. Families, physicians, and dentists exert pressure on manufacturers to produce sugar‐free alternatives, writing down the full content of their solutions (especially sugar content and pH) and the main preventive habits.

Various professionals and parties are involved in the manufacturing, prescribing, and utilization of PLMs in children. The correct interplay and collaboration between these actors are crucial in minimizing the oral health side effects of PLMs. Despite this, we could not find any case study on this synergy between healthcare professionals, families, and manufacturers aiming at reducing the risks of dental caries among children on PLMs. Figure 5 illustrates our proposed model for a successful collaboration between the parties.

In fact, several preventive case studies have been implemented using interdisciplinary approaches and collaboration between several parties with promising results. For instance, US researchers assessed the design, implementation, and early results of North Carolina preventive dental programs for low‐income 0–35‐month‐olds. The primary focus was the Medicaid‐funded “Into the Mouths of Babes” program, which reimbursed pediatricians, family physicians, and community health practitioners for preventative dental care. These services included risk assessment, screening, fluoride varnish, referrals, and caregiver counseling. Clinicians received continuous medical education through lectures, interactive workshops, practice guidelines, case‐based challenges, practical procedures, a resource toolkit, and follow‐up training. In the first 2 years, 1595 doctors were trained. Results demonstrated that non‐dental caregivers may provide preventive dental care to young Medicaid‐enrolled children without dentists. The study showed that medical practices may provide preventative dental care, especially in poor communities, and that healthcare personnel need training to treat ECC [48].

Another study investigated and piloted an iPad‐based ECC risk assessment and mitigation program for low‐income Latino families. My Smile Buddy (MSB), designed by a multidisciplinary team, helped Community Health Workers (CHWs) assess ECC risk and encourage behavioral changes through interactive components and motivating interviews. Five CHW and community focus groups evaluated content, practicality, functionality, design, and effectiveness during development. A “diet widget” and risk algorithm assessed diet and ECC risk in the app. Health behavior change planning with parents emphasized community‐based participatory research. In pilot testing, mothers (*n* = 35) and CHWs rated MSB's usability and utility as high. The study suggested power‐sharing in intervention creation to make it engaging and community‐accepted. The study discovered ECC risk factors include maternal dental caries history, frequent sugar exposures, insufficient fluoride exposure, high plaque scores, and increased salivary *Streptococci Mutans* levels [49].

A case study explored how pediatric interprofessional education in clinical experience using oral‐systemic health benefited family nurse practitioner, dental, and medical students' self‐reported interprofessional competence. Students learned to assess pediatric oral health, discover the oral‐systemic connection, and collaborate to improve outcomes through the interprofessional experience. In an outpatient clinic, a pediatric dental resident and 162 family nurse practitioners, dentistry, and medical students interacted. Staff reviewed the patient's chart, took a medical and dental history, checked oral health, applied fluoride varnish, and offered information and anticipation. Results indicate significant gains in mean scores from pretest to posttest for all students, including nurse practitioner (*p* < 0.01), dentistry (*p* < 0.01), and medicine (*p* < 0.001). The pre‐ to posttest changes were considerable in pediatric dental and primary care settings (*p* < 0.001). The event boosted interprofessional teamwork in all student groups [50].

These studies underscore the potential of interprofessional collaborative approach between community health initiatives to address clinical problems and pediatric health disparities. Healthcare professionals and families can be empowered to proactively manage health risks through personalized, culturally relevant interventions.

### 3.9 Preventive Measures

In the light of the previous data and information, the following preventive recommendations can be made:

– Children on PLMs for a long duration should be monitored regularly to make the diagnosis and treatment of caries/erosion as fast and efficient as possible. These children should be motivated and instructed to practice regular oral hygiene measures.
– The use of sugar‐free PLMs must be suggested whenever possible.
– Drops are preferred over syrups as the risk of dentition involvement is lower.
– Due to slower oral clearance at night, patients should be urged to take their medications only in mealtimes and to avoid taking them right before bedtime.
– After consuming PLMs, children should be advised to wash their mouths with water; however, immediate brushing should be avoided. Chewing sugar‐free gums may also be helpful.
– Manufacturers should acknowledge the sugar content and pH of PLMs on their labels clearly. Also, statements such as “this medication may be harmful to the teeth” are recommended to be written on the labels.
– Cooperation between dentists, physicians, pediatricians, and families is crucial. Therefore, these parties should be acknowledged about the potential harmful effects of PLM on the dentition and the preventive methods.

To facilitate the adoption of these preventive measures and make the recommendations more universally applicable, especially in resource‐limited settings, the following strategies can be applied:

- Advocacy and policy support: Encouraging healthcare providers and policymakers to promote the availability and affordability of sugar‐free PLMs through subsidies or inclusion in essential medicines lists.
- Education and awareness campaigns: Conducting community‐based education to inform families about the benefits of sugar‐free medications and how to request them from healthcare providers or pharmacies.
- Collaboration with pharmacists and suppliers: Working with local pharmacies and drug suppliers to prioritize stocking and recommending sugar‐free formulations, possibly through incentive programs.
- Utilizing local resources: Promoting the use of locally available, affordable alternatives such as natural flavorings or formulations that do not contain added sugars, where commercially available options are limited.
- Training healthcare providers: Providing training for dentists, physicians, pediatricians, and pharmacists on the importance of prescribing and dispensing sugar‐free PLMs and on effective communication strategies with families.
- Patient and caregiver engagement: Empowering families with information on how to identify and request sugar‐free options, and how to implement other preventive practices effectively.

Including these strategies would make the preventive recommendations more practical and achievable across diverse settings, ensuring that children everywhere can benefit from safer medication options and better oral health outcomes.

### 3.10 Strength and Limitations

The strength point of this review is its broad scope, which includes nearly all the study designs on the association investigated, from in vitro and in vivo cross‐sectional studies to epidemiological and longitudinal ones. Moreover, not only the mechanical and biological relation between PLMs and dental caries, but also the role of dentists, physicians, pediatricians, pharmacists, families, and manufacturers has been discussed. Besides, clinical implications and practical preventive measures have also been included. However, narrative reviews bear some limitations by their nature in terms of a complete literature search, objectivity, and interpretation of the results. By applying the PRISMA guideline and conducting a semi‐systematic search in multiple databases, we tried to overcome the study selection bias. Team discussion was used to minimize the bias that may arise from objectivity and interpretation issues.

## 4 Conclusion

This narrative review aimed to provide a comprehensive update on PLM consumption as a risk factor for dental caries, especially in chronically ill children. Physiochemical properties of PLM solution, such as their sugar content and endogenous pH define their carcinogenicity and erosive potential. Indeed, several in vitro studies report the erosive potential of PLMs on dental hard tissue structure when viewed under scanning electron microscopes. Furthermore, both in vivo and epidemiological studies support this association and suggest a higher caries risk experience among chronically ill children on PLMs. Finally, this review provided some preventive measures and recommends a model for positive collaboration between pediatricians, dentists, and families aiming to maximize the benefits of PLMs and minimizing its possible harms on oral health.

## Author Contributions

**Mohammed Taib Fatih:** visualization, methodology, investigation, software, conceptualization. **Mohammed Khalid Mahmood:** conceptualization, investigation, writing – original draft, methodology, writing – review and editing, formal analysis. **Handren Ameer Kurda:** validation, software, investigation. **Herve Tassery:** conceptualization, methodology, project administration, supervision. **Romain Lan:** conceptualization, investigation, writing – original draft, methodology. **Delphine Tardivo:** conceptualization, supervision, project administration. **Mohammed Aso Abdulghafor:** visualization, data curation, methodology, investigation.

## Conflicts of Interest

The authors declare no conflicts of interest.

## Transparency Statement

The lead author, Mohammed Khalid Mahmood, affirms that this manuscript is an honest, accurate, and transparent account of the study being reported; that no important aspects of the study have been omitted; and that any discrepancies from the study as planned (and, if relevant, registered) have been explained.

## Data Availability

The data that support the findings of this study are available from the corresponding author upon reasonable request.
